# Comprehensive analysis of the *GeBP* gene family in cucumber highlights functional divergence in fruit development and stress tolerance

**DOI:** 10.1186/s12870-026-08774-6

**Published:** 2026-04-18

**Authors:** Xing Wang, Zixuan Zhao, Chang Liu, Nanyang Li, Liping Wang, Suna Wang, Kaijing Zhang, Junjun Cui

**Affiliations:** 1 School of Landscape and Ecological Engineering, Hebei University of Engineering, Handan, 056038 China; 2 College of Agriculture, Anhui Science and Technology University, Chuzhou, 233100 China

**Keywords:** CsGeBP, high expression, fruit development, stress responses

## Abstract

**Background:**

*GeBP* genes play important roles in plant development and stress responses; however, they have not yet been systematically characterized in cucumber (*Cucumis sativus*).

**Results:**

In this study, six *GeBP* genes were identified in the cucumber genome. All CsGeBP proteins possess conserved basic regions and leucine zipper–like domain and are predominantly localized in the nucleus. Phylogenetic and synteny analyses revealed that the *GeBP* gene family is highly conserved among cucurbit species and has expanded mainly through segmental duplication events. Expression analyses demonstrated clear functional divergence among *CsGeBP* genes. *CsGeBP5* showed consistently high expression in reproductive tissues and was upregulated in long-fruit cucumber lines, suggesting a potential role in fruit development. In contrast, *CsGeBP6* was strongly induced under salt stress, and its expression was further enhanced by silicon treatment. Moreover, it was markedly upregulated in the resistant cucumber line SSL508-28 after powdery mildew inoculation, suggesting a role in both abiotic stress tolerance and disease resistance.

**Conclusions:**

Overall, this study provides the first comprehensive analysis of the *GeBP* gene family in cucumber and identifies key potential genes associated with fruit development (*CsGeBP5*) and stress responses (*CsGeBP6*), laying a foundation for future functional studies and molecular breeding.

**Supplementary Information:**

The online version contains supplementary material available at 10.1186/s12870-026-08774-6.

## Background

Glabrous Enhancer Binding Proteins (GeBPs) constitute a plant-specific transcription factor family distinguished by a non-canonical basic region–leucine zipper (bZIP-like) domain that confers DNA-binding activity [1]. The first member was identified in *Arabidopsis thaliana*, where it binds to the enhancer of *GLABROUS1* (*GL1*), a key regulator of trichome development, to regulate leaf primordium differentiation and cell expansion. This discovery highlighted the unique role of *GeBPs* in promoting cell enlargement rather than cell proliferation [2].

GeBP proteins predominantly localize to the nucleus and exert their biological functions by modulating the transcription of downstream target genes. The advent of high-throughput genome sequencing has facilitated systematic exploration of this family across diverse plant species. Cumulative evidence demonstrates that *GeBPs* participate not only in developmental regulation but also in phytohormone-mediated signaling and diverse environmental stresses. For instance, nine *GmGeBP* genes have been identified in soybean, with conserved gene structures and tissue-specific expression patterns; *GmGeBP4* is highly expressed in aberrant trichomes, suggesting its involvement in epidermal patterning [3]. In monocot crops, 125 *GeBP* members have been identified and classified into four clades, with whole-genome and segmental duplications serving as major drivers of gene family expansion. Most monocot *GeBPs* exhibit predominant expression in photosynthetic and floral tissues and display robust transcriptional responsiveness to hormonal and heavy metal stress signals [4].

Mechanistic studies have revealed that *GeBP/GPL* genes are intricately integrated into developmental regulatory networks. The prototype *GeBP* is directly regulated by KNAT1, a KNOTTED-like homeobox transcription factor essential for shoot apical meristem maintenance and plant morphology [2]. KNOX proteins promote cytokinin biosynthesis, and subsequent work demonstrated that GeBP and GPL proteins serve as key regulators of the cytokinin signaling pathway [1], positioning the *GeBP/GPL* family as a central molecular hub integrating developmental and hormonal cues. Recently, Zhu et al. proposed that GeBP proteins also possess transmembrane and major facilitator superfamily (MFS) transporter-like features, implying their involvement in transmembrane signal perception during environmental challenges [5]. Post-transcriptional regulation further modulates *GeBP* activity. *miR827*, induced by leaf senescence and phosphate starvation, represses *GPLa*, leading to upregulation of phosphate transporter genes (*PHT1*) and enhanced phosphorus accumulation. Conversely, *GPLa* overexpression suppresses *PHT1* expression and reduces phosphorus content, demonstrating that *GPLa* functions as a negative regulator of phosphate uptake and transport [6]. In addition, the *GeBP/GPL* family plays conserved roles in responses to abiotic and biotic stresses. Genome-wide analyses in *Brassica rapa* and *Malus domestica* reported strong induction of specific *GeBP* members under drought and cold conditions, implicating them as core stress-responsive transcriptional regulators [7, 8]. Moreover, Tobacco curly shoot virus (TbCSV) βC1 protein has been shown to specifically bind Solanaceae GeBPs, representing a previously unrecognized virus–host interaction strategy in which viral proteins reprogram host transcriptional networks to enhance infection [9].

Cucumber (*Cucumis sativus L.*) is an economically important vegetable crop worldwide, yet its growth and productivity are increasingly constrained by drought, heat stress, salinity, and other environmental challenges. Despite the emerging recognition of the multifaceted roles of *GeBPs* in development and stress adaptation, the *GeBP* gene family has not been systematically characterized in cucumber. In recent years, with the release and continuous improvement of the cucumber reference genome [10, 11], significant progress has been made in genome-wide identification and functional prediction of gene families. More and more cucumber varieties, including East Asian and European greenhouse types, are being used in genomic and transcriptomic studies [12, 13]. In this context, a systematic identification and expression analysis of the *GeBP* gene family will not only provide valuable candidate genes for functional studies but also lay a theoretical foundation for subsequent functional validation across different genetic backgrounds.

## Methods

### Identification of the *GeBP* Gene family in cucumber

The Hidden Markov Model (HMM) of the *GeBP* gene family (Pfam ID: PF04504) was retrieved from the Pfam database (http://pfam.xfam.org/). The cucumber genome sequences were downloaded from the *Cucurbitaceae* Genomic Database (http://cucurbitgenomics.org) [10]. Candidate *GeBP* family members were identified in the cucumber genome using HMMER v3.0 with the default parameters [14]. Protein sequences with an E-value < 1 × 10⁻⁵ were retained as high-confidence candidates and extracted using Perl scripts.

All candidate GeBP protein sequences were aligned using ClustalW to generate a cucumber-specific HMM profile, which was subsequently used to perform a second-round genome-wide search for *GeBP* genes. All putative GeBP proteins were further validated using the NCBI Conserved Domain Database (CDD) (http://www.ncbi.nlm.nih.gov/Structure/cdd/wrpsb.cgi) to confirm the presence of the conserved GeBP domain [15].

*GeBP* family members in other cucurbit crops, including melon (*Cucumis melo* L.), wax gourd (*Benincasa hispida*), and watermelon (*Citrullus lanatus*), were identified using the same procedure. *GeBP* genes from wheat (*Triticum aestivum*), rice (*Oryza sativa* subsp. *indica*), soybean (*Glycine max*), and *Arabidopsis thaliana* were obtained from the PlantTFDB database (http://planttfdb.gao-lab.org/).

### Characterization and phylogenetic analysis of GeBP proteins

The physicochemical properties of GeBP proteins, including amino acid length, molecular weight (MW), theoretical isoelectric point (pI), instability index, aliphatic index, and grand average of hydropathicity (GRAVY), were calculated using ExPASy ProtParam tool (https://web.expasy.org/protparam/) [16]. Subcellular localization was predicted using the CELLO v2.5 server (http://cello.life.nctu.edu.tw/).

Conserved motifs of GeBP proteins were identified using MEME Suite (http://meme-suite.org/), with the maximum number of motifs set to 10 and the motif width ranging from 6 to 200 amino acids. Multiple sequence alignment was conducted using ClustalX 2.0, and the alignment results were visualized with Jalview [17]. A phylogenetic tree was constructed using MEGA 7.0 [18] with the neighbor-joining (NJ) method under the Poisson substitution model, pairwise deletion of gaps, and 1000 bootstrap replications [19].

### Chromosomal localization and synteny analysis of *GeBP* Genes

The physical positions of *GeBP* genes were obtained from the genome annotation files and mapped onto cucumber chromosomes using MapChart [20]. The chromosomal distribution of *GeBP* genes in cucurbit crops was visualized using TBtools [21]. Gene duplication events, including segmental duplications (defined as gene pairs located on different chromosomes or on the same chromosome but not adjacent and identified within syntenic blocks by MCScanX) and tandem duplications (defined as gene pairs located on the same chromosome, adjacent to each other or separated by no more than one intervening gene and meeting the BLASTP similarity threshold of E-value < 1 × 10⁻⁵), were identified using MCScanX [22]. Synteny relationships among *GeBP* genes in different cucurbit species were visualized using the Dual Synteny Plotter module implemented in TBtools (https://github.com/CJ-Chen/TBtools) [23].

### Cis-acting regulatory element analysis of *CsGeBP* genes

The 1,500 bp upstream sequences from the transcription start site of each *CsGeBP* gene were defined as promoter regions and extracted using TBtools. The promoter sequences were submitted to the PlantCARE database (http://bioinformatics.psb.ugent.be/webtools/plantcare/html/) for the identification of cis-acting regulatory elements.

### Tissue-specific expression analysis of *CsGeBP* genes in cucumber

RNA-seq data with accession number PRJNA80169 and PRJNA258122 [24, 25] were obtained from the NCBI Sequence Read Archive (SRA). The SRA files were first converted to FASTQ format using the fasterq-dump.2.11.0 (https://github.com/ncbi/sra-tools/wiki/HowTo:-fasterq-dump). Raw sequence quality was assessed with the FastQC plugin. Adapter sequences and low-quality reads were removed using the Trimmomatic plugin, resulting in high-quality clean reads after preprocessing. The filtered reads were then aligned to the Chinese Long_V3 reference genome using STAR to generate BAM files. Gene expression levels were subsequently quantified with the StringTie-Quantify plugin. Finally, differentially expressed genes (DEGs) were identified using the DESeq2 plugin based on normalized read counts. The expression patterns of *CsGeBP* genes were analyzed in leaf, stem, female flower, male flower, unfertilized ovary, fertilized ovary, ovary, root, tendril, and tendril base. The expression levels of *CsGeBP* genes were visualized as heatmaps using TBtools.

### Expression analysis of *CsGeBP* genes under biotic and abiotic stresses

RNA-seq datasets PRJNA634519 [26], PRJNA438923 [27], PRJNA477930 [28], PRJNA437579, PRJNA285071 [29], PRJNA321023 [30], and PRJNA419665 [31] were downloaded from the NCBI SRA database. These datasets were used to analyze the expression profiles of *CsGeBP* genes under high-temperature stress, low-temperature stress, salt stress and silicon (Si) treatment, downy mildew infection, powdery mildew infection, and root-knot nematode infection. Heatmaps illustrating the expression patterns of *CsGeBP* genes in response to biotic and abiotic stresses were generated using TBtools.

### Salt stress treatment and qRT-PCR analysis

Cucumber cultivar ‘Jinyan-4’ (provided by the Tianjin Kerun Agricultural Technology Stock Co.,Ltd. Vegetable Research Institute) was used as the experimental material. Seeds were germinated and seedlings were grown under controlled environmental conditions. Uniform and healthy seedlings (two-leaf and one-heart stage) were selected for salt stress treatment. All plants were divided into three groups: (i) control plants irrigated with clean water, (ii) plants treated with 75 mM NaCl, and (iii) plants treated with 75 mM NaCl supplemented with 0.5 mM organic silicon (tetraethyl orthosilicate, Shanghai Silicon Mountain Polymer Materials Co., Ltd. (Shanghai, China)). the leaves of the seedlings were removed at 0, 3, 5 and 12 days after treatment for qRT‒PCR. Total RNA was extracted from frozen leaf tissues using a plant RNA extraction kit (DP432; Tiangen Biotech, Beijing, China) according to the manufacturer’s instructions. Specific primers for each gene were designed through Primer 6 (Table 1). The synthesized cDNA was subjected to qRT-PCR analysis using an Opticon thermocycler (CFX96 Connect Real-Time System; Bio-Rad, Hercules, CA, USA) with SYBR Green PCR Master Mix (Vazyme, Nanjing, China), according to the manufacturer’s instructions. Relative expression levels were calculated using the 2^⁻ΔΔCt^ method [32], and data analysis was performed using Microsoft Excel.

**Table 1:** List of primers used in quantitative RT‒PCR, which designed with primer 6

gene	Sense Primer	Anti-sense Primer
*CsGeBP6*	GAAGAAGAAGAAGAGGATGAAG	TGAGCCTGAGAGTTAGTAGA
*Csa6G484600(Actin)*	CTGGTGATGGTGTGAGTC	AGAGATGGCTGGAATAGAAC

## Results

### Identification and physicochemical characterization of the *GeBP* gene family in cucumber

**Fig. 1:**
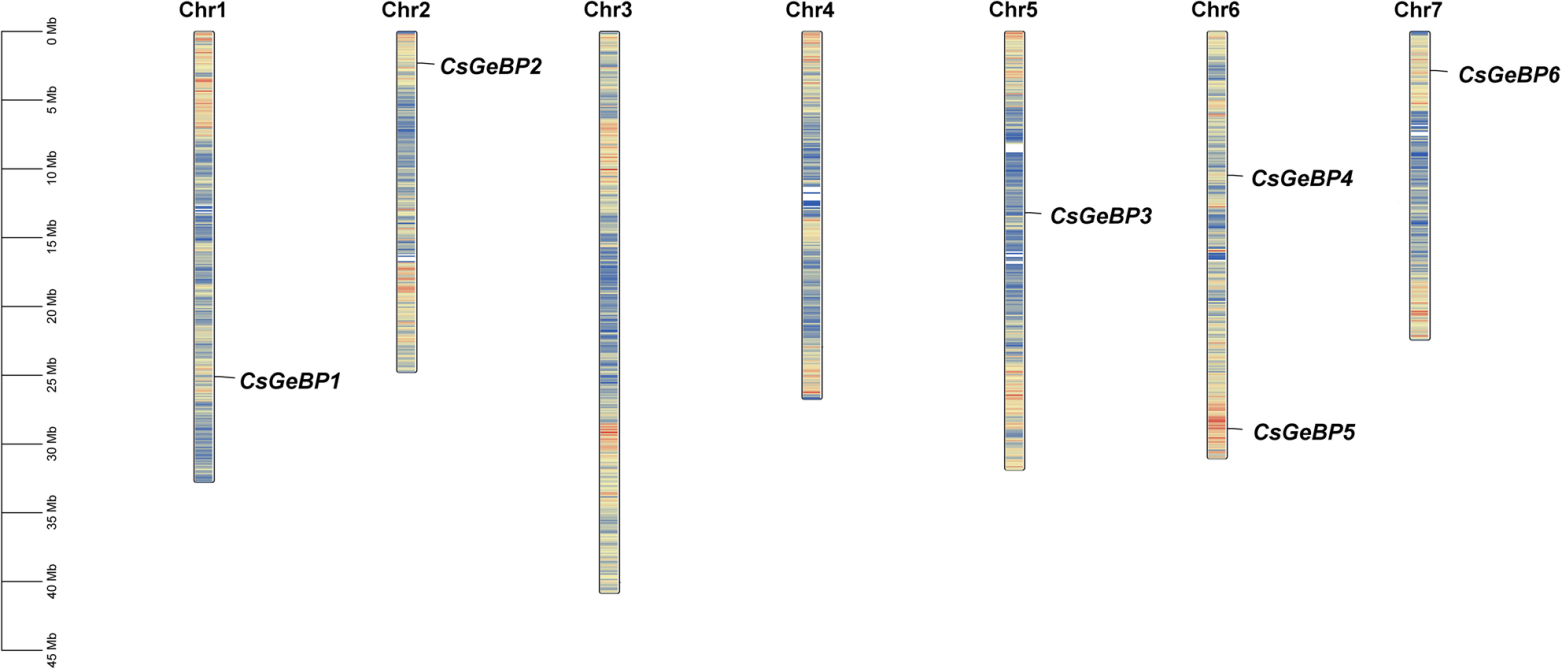
Distribution of *CsGeBP* family genes on the chromosomes of cucumber

A total of six members of the *GeBP* gene family were identified in the cucumber genome and were sequentially named *CsGeBP1*-*CsGeBP6* according to their chromosomal locations (Fig. 1). All candidate proteins were subjected to domain validation, and it was confirmed that each contained a typical conserved GeBP domain. These genes are distributed across five of the seven cucumber chromosomes, with two GeBP genes located on chromosome 6 and one on each of the remaining chromosomes, except chromosomes 3 and 4, on which no GeBP genes were detected. Sequence analysis showed that the predicted proteins ranged from 179 amino acids (*CsGeBP3*) to 419 amino acids (*CsGeBP5*), with molecular weights of 21.19 kDa (*CsGeBP3*) to 46.76 kDa (*CsGeBP6*). The predicted isoelectric points (*pI*) varied from 5.10 (*CsGeBP3*) to 10.11 (*CsGeBP4*); among them, *CsGeBP3* and *CsGeBP4* were basic proteins, whereas the remaining members were acidic proteins. The aliphatic index ranged from 57.09 (*CsGeBP5*) to 84.65 (*CsGeBP4*), and the instability index ranged from 38.24 (*CsGeBP3*) to 60.33 (*CsGeBP6*), indicating that most CsGeBP proteins are unstable (instability index > 40). The grand average of hydropathicity (GRAVY) values ranged from − 1.107 (CsGeBP6) to -0.723 (CsGeBP4), suggesting that all CsGeBP proteins are hydrophilic. Subcellular localization prediction revealed that six *CsGeBP* genes were localized in the nucleus, while *CsGeBP4* and *CsGeBP5* were also predicted to be Cytoplasmic (Table 2).

**Table 2:** Physicochemical properties of *CsGeBP* gene family members

Species	Gene ID	Name	Number of amino acids (aa)	Molecular weight (D)	pI	Instability index	Aliphatic index	Average of hydropathicity	Prediction of subcellular location
*Cucumis sativus* L.	*CsaV3_1G039710.1*	*CsGeBP1*	402	45144.15	5.10	50.18	72.79	-0.819	Nucleus
	*CsaV3_2G004350.1*	*CsGeBP2*	370	42548.30	5.26	56.63	74.11	-0.979	Nucleus
	*CsaV3_5G015720.1*	*CsGeBP3*	179	21194.25	9.50	38.24	75.14	-1.040	Nucleus
	*CsaV3_6G014420.1*	*CsGeBP4*	228	26151.82	10.11	57.19	84.65	-0.723	Nucleus/‌Cytoplasmic
	*CsaV3_6G049420.1*	*CsGeBP5*	419	46093.38	5.78	49.16	57.09	-1.049	Nuclear/Cytoplasmic
	*CsaV3_7G003860.1*	*CsGeBP6*	404	46755.06	5.24	60.33	67.30	-1.107	Nucleus

### Gene structure and conserved motif analysis of the *GeBP* gene family in cucumber

**Fig. 2:**
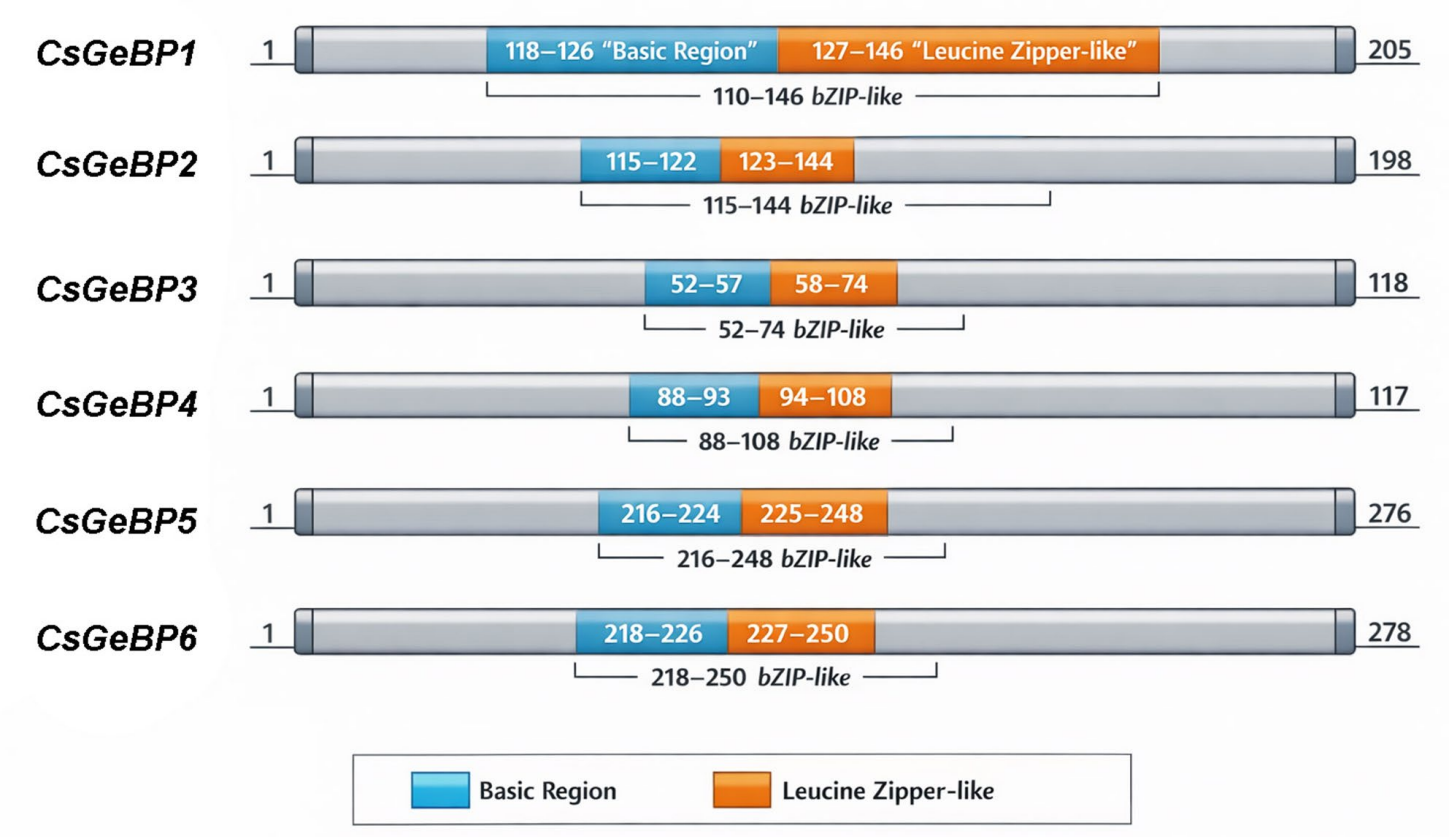
Conserved domains of CsGeBP proteins

DNAMAN-based multiple sequence alignment revealed that the CsGeBP proteins possess highly conserved domains (Fig. 2). Notably, all CsGeBP proteins contain a basic region and a leucine zipper–like motif, indicating a high degree of structural conservation within the family.

**Fig. 3:**
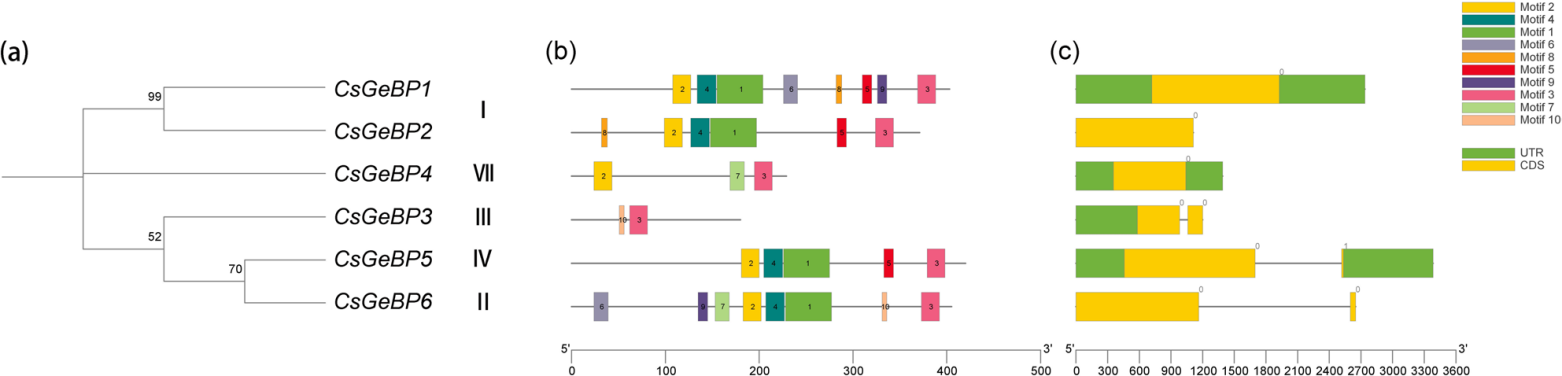
Phylogenetic tree, gene structure, and conserved domains of the *CsGeBP* genes in cucumber. **a** The phylogenetic tree of CsGeBP proteins, with different colors representing distinct clades. **b** Conserved domain alignment of CsGeBP proteins. **c** Gene structure of *CsGeBP* genes, in which yellow boxes represent coding sequences (CDS), green boxes indicate untranslated regions (UTRs), and black lines denote introns

Phylogenetic analysis grouped the six CsGeBP proteins into five clades (Fig. 3a). Conserved motif analysis revealed that CsGeBP1 and CsGeBP6 contain multiple highly conserved core motifs (such as Motifs 1, 2, 3, and 4). CsGeBP3 possesses relatively fewer motifs but still contains the typical conserved core GeBP motifs. CsGeBP4 harbors a complete set of core conserved motifs, and its motif arrangement is similar to that of CsGeBP1. CsGeBP5 contains multiple core motifs as well as some specific motifs, with a relatively complete domain distribution. Overall, all CsGeBP proteins contain the typical conserved core motifs (Motif 3) of the GeBP family (Fig. 3b). Most *CsGeBP* genes consisted of one or two exons (Fig. 3C), suggesting a conserved evolutionary pattern among *CsGeBP* family members, while differences in motif number and composition may indicate potential functional diversification.

### Phylogenetic analysis of the *GeBP* family

**Fig. 4:**
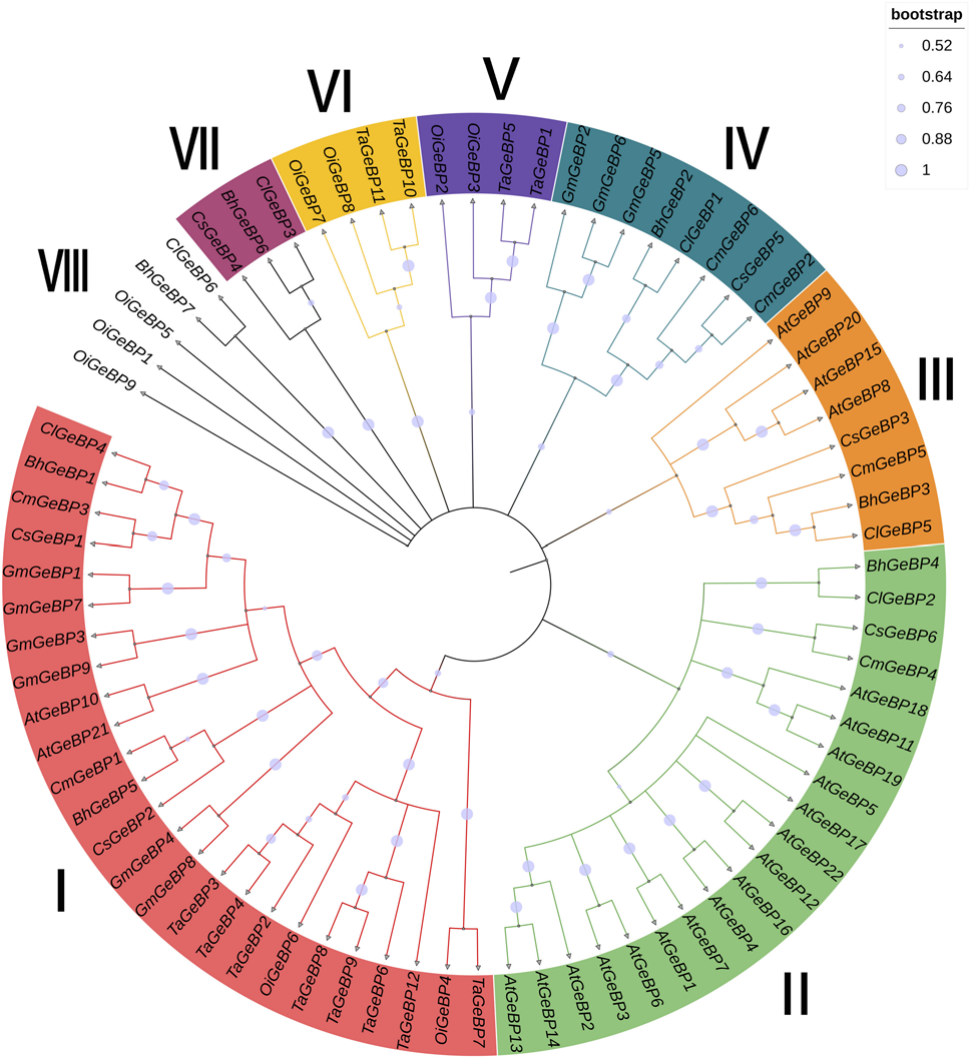
Phylogenetic tree of *GeBP* family members in *(A) thaliana*, *C. sativus*, *C. melo*, *C. lanatus*, *(B) hispida*, *G. max*, *O. sativa subsp. indica* and *T. aestivum*

To elucidate the evolutionary relationships of the *GeBP* family, a phylogenetic tree was constructed using full-length GeBP protein sequences from *(A) thaliana*, *C. sativus*, *C. melo*, *C. lanatus*, *(B) hispida*, *G. max*, *O. sativa subsp. indica* and *T. aestivum* (Fig. 4; Table S1). The results showed that GeBP proteins were clearly classified into eight distinct subgroups (Groups I–VIII). As shown in the Fig. 4, the nodes of the major clades (Groups I–VIII) are supported by relatively high bootstrap values. Most of the key divergence nodes show bootstrap support values ranging from 0.76 to 1.0, with some core branches approaching 1, indicating that the subgroup classification has strong statistical reliability. Group I represented one of the largest clades and contained *GeBP* members from all examined species. Multiple paralogous gene pairs from cucurbit crops and soybean were observed within this subgroup, suggesting that Group I experienced ancient duplication events followed by lineage-specific expansion. Group II was characterized by a relatively high number of *Arabidopsis GeBP* members, together with homologs from cucurbit species. The close clustering of orthologous genes from different species implies strong evolutionary constraints acting on this subgroup. No dicotyledonous members were detected in Group V or Group VI in the phylogenetic tree, further supporting that lineage-specific evolutionary diversification of the *GeBP* family occurred after the divergence of monocotyledonous and dicotyledonous plants. Collectively, this phylogenetic analysis provides an evolutionary basis for subsequent comparative and functional investigations of *GeBP* genes in cucurbit species.

**Fig. 5:**
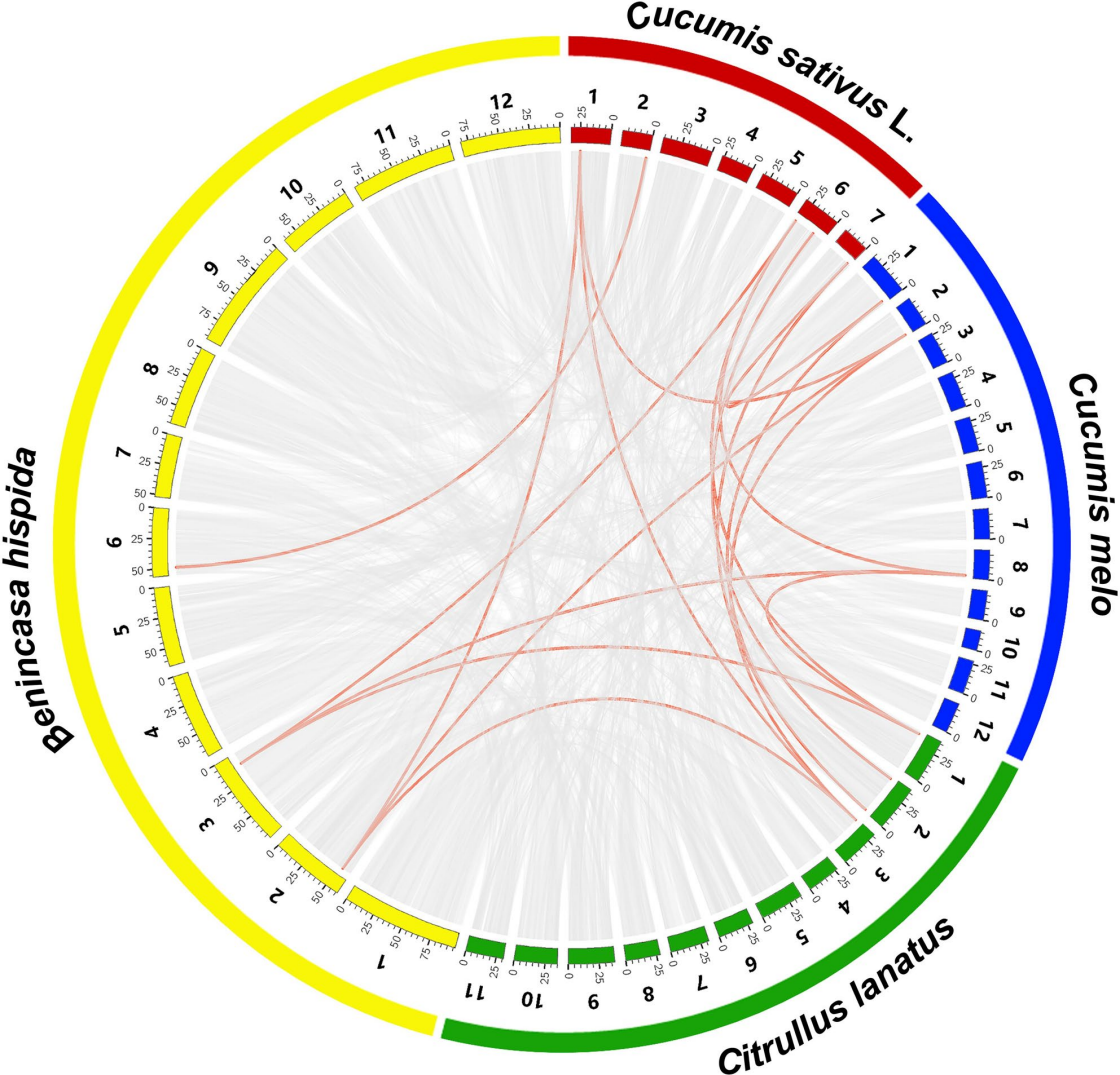
Syntenic analysis of *CsGeBP* genes among *C. sativus*, *C. melo*, *C. lanatus*, and *B. hispida*. Gray lines in the background represent collinear genomic blocks within and between the four species, whereas red lines indicate syntenic *CsGeBP* gene pairs

The subgroup-specific expansion patterns observed in the phylogenetic tree were further supported by collinearity analysis, which revealed multiple segmental duplication events within cucurbit *GeBP* genes (Fig. 5; Table 3). Numerous syntenic *GeBP* gene pairs were identified among these species, indicating that *GeBP* genes are highly conserved across cucurbit crops. Most syntenic *GeBP* gene pairs were located on different chromosomes rather than forming tandem duplications, suggesting that the expansion of the *GeBP* family in cucurbits was predominantly driven by segmental duplication or whole-genome duplication events. Notably, cucumber and melon shared a greater number of syntenic *GeBP* gene pairs compared with watermelon and wax gourd, reflecting their closer evolutionary relationship and a higher degree of chromosomal collinearity.

**Table 3:** Collinear gene pairs of *GeBP* genes present in the four Cucurbitaceae genomes

Number	Cucumber	Melon	Watermelon	Waxgourp
1	*CsaV3_1G039710*	*MELO3C015400P1*	*Cla97C03G066530*	*Bhi02G001643*
2	*CsaV3_2G004350*	*MELO3C004359P1*	*-*	*Bhi06G001498*
3	*CsaV3_6G014420*	*-*	*Cla97C02G047710*	*-*
4	*CsaV3_6G049420*	*MELO3C007351P1*	*Cla97C01G022150*	*Bhi03G000396*
5	*CsaV3_7G003860*	*MELO3C018793P1*	*Cla97C02G028080*	*-*

### Cis-acting element analysis of *CsGeBP* genes

**Fig. 6:**
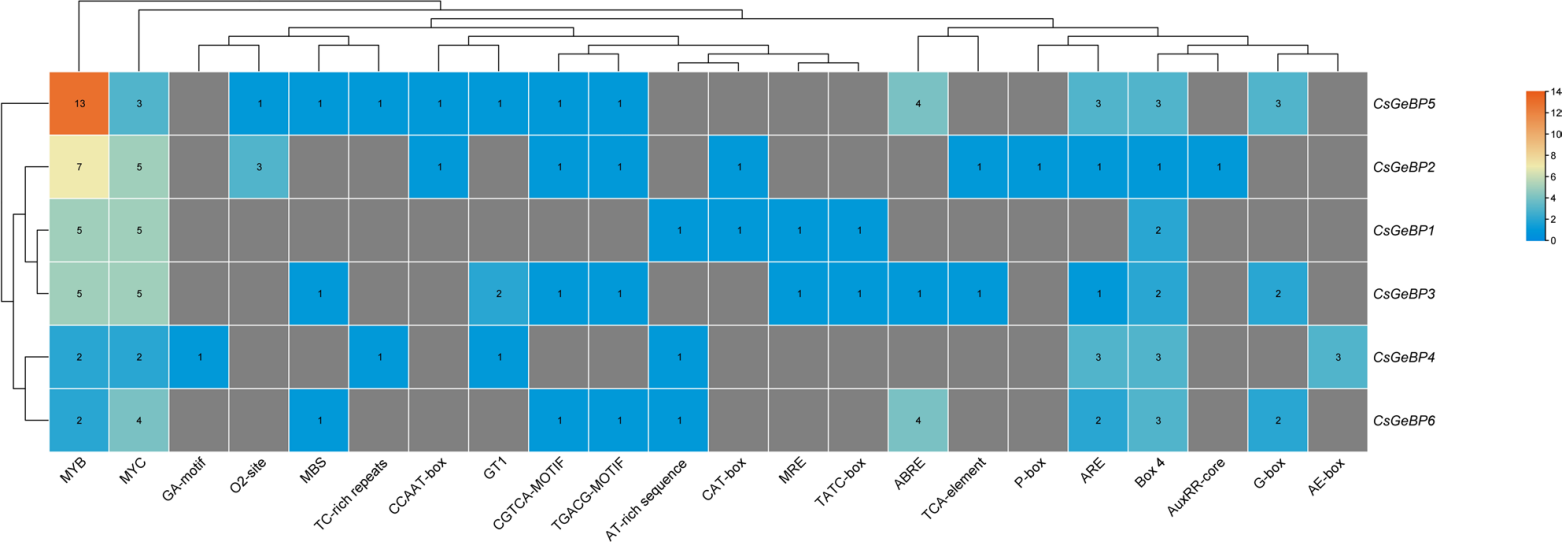
Heatmap of cis-acting elements in the promoter regions of *CsGeBP* genes. Color intensity indicates the abundance of cis-elements, with deeper red representing higher numbers

Cis-acting element analysis of the 1.5 kb promoter regions of *CsGeBP* genes revealed the widespread presence of stress- and hormone-responsive elements (Fig. 6). Key cis-elements associated with abiotic stress and hormone signaling, including MYB, MYC, MBS, ABRE, ARE, and G-box, were commonly identified across *CsGeBP* promoters, suggesting that *CsGeBP* genes may possess predicted regulatory potential in stress- and hormone-mediated regulatory pathways. Notably, the composition and abundance of cis-elements varied among *CsGeBP* genes. *CsGeBP5* harbored the highest number and diversity of cis-elements, whereas other members exhibited relatively simpler promoter architectures. Hierarchical clustering further indicated divergence in cis-element distribution patterns among *CsGeBP* promoters, implying functional differentiation at the transcriptional regulation level.

### Expression patterns of *CsGeBP* genes in different tissues

**Fig. 7:**
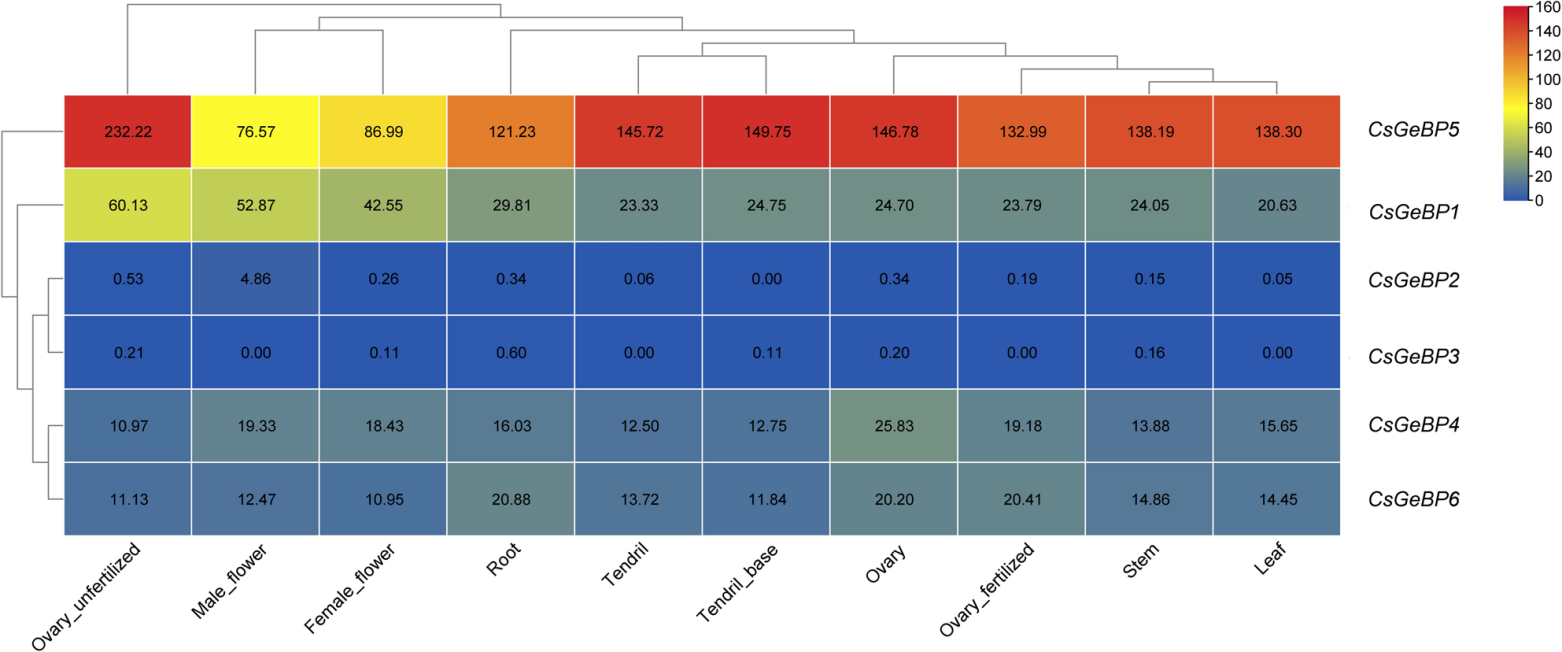
Expression heatmaps of *CsGeBP family* genes in different tissues of cucumber

Transcriptome-based expression analysis revealed distinct tissue-specific expression patterns among *CsGeBP* genes (Fig. 7). *CsGeBP5* (*CsaV3_6G049420*) exhibited consistently high expression levels across all examined tissues, with particularly strong expression in ovary-related tissues and tendrils, suggesting a potential role in reproductive development and vegetative growth. In contrast, several *CsGeBP* genes displayed moderate and relatively stable expression across tissues, whereas others showed very low expression levels. Hierarchical clustering further classified *CsGeBP* genes into high-, moderate-, and low-expression groups, indicating functional diversification within the *GeBP* gene family at the transcriptional level.

**Fig. 8:**
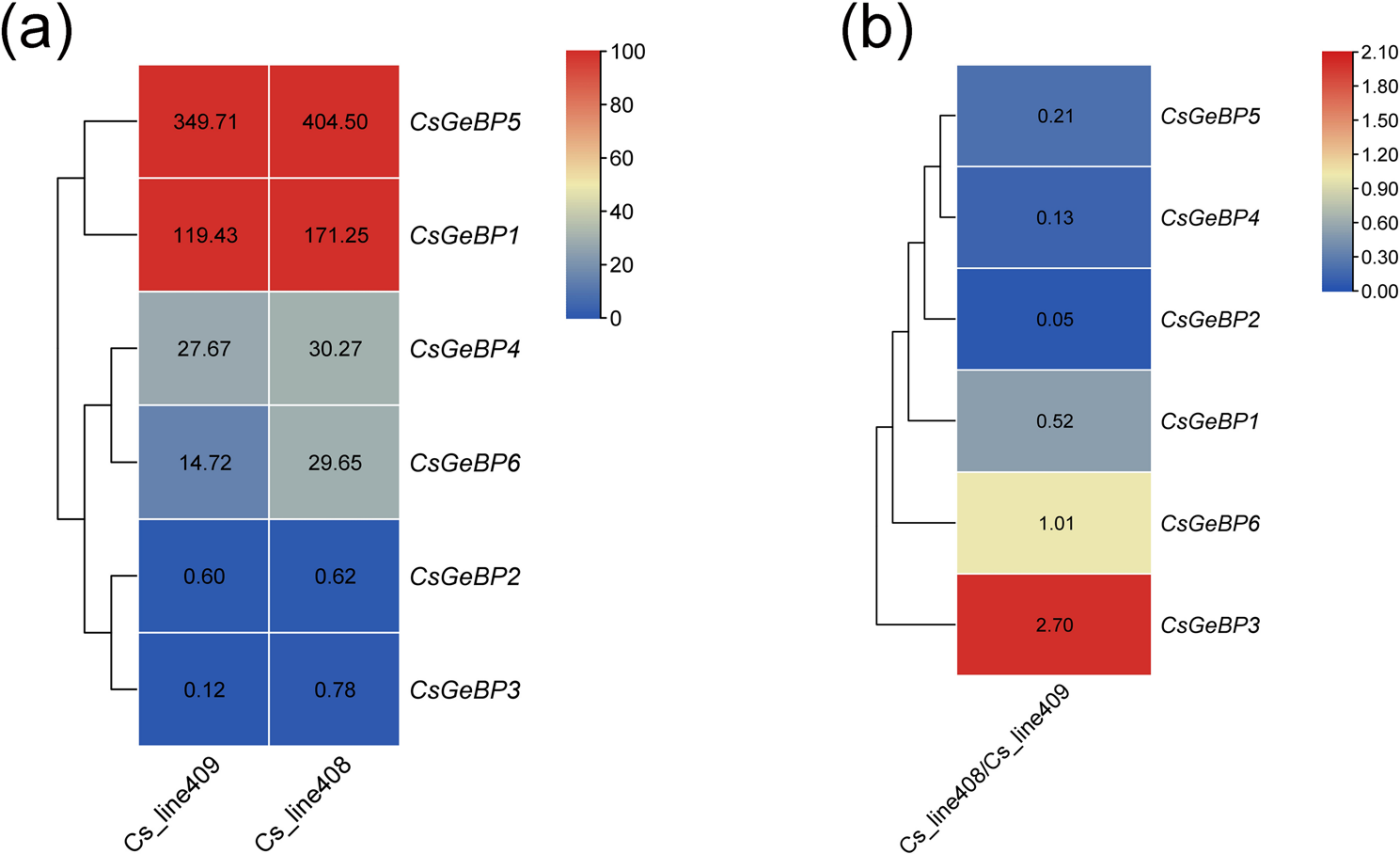
Expression heatmaps of *CsGeBP* genes in the long-fruit line (Cs_line408) and the short-fruit line (Cs_line409). **a** The data in the table represent the raw FPKM values. **b** The data in the table represent the log2 FC values

Consistent with their tissue-specific expression patterns, *CsGeBP* genes also showed differential expression during early fruit development between the long-fruit line (Cs_line408) and the short-fruit line (Cs_line409). *CsGeBP5* and *CsGeBP1*, which are highly expressed in reproductive tissues, were further upregulated in long fruit, whereas *CsGeBP3* and *CsGeBP6* exhibited pronounced log₂ fold changes, indicating that they should be considered candidate regulators in fruit length determination (Fig. 8).

### Expression analysis of *CsGeBP* genes under abiotic and biotic stresses

Based on public NCBI SRA transcriptome data, the expression patterns of cucumber *CsGeBP* genes under biotic and abiotic stresses were analyzed and visualized using heat maps.

**Fig. 9:**
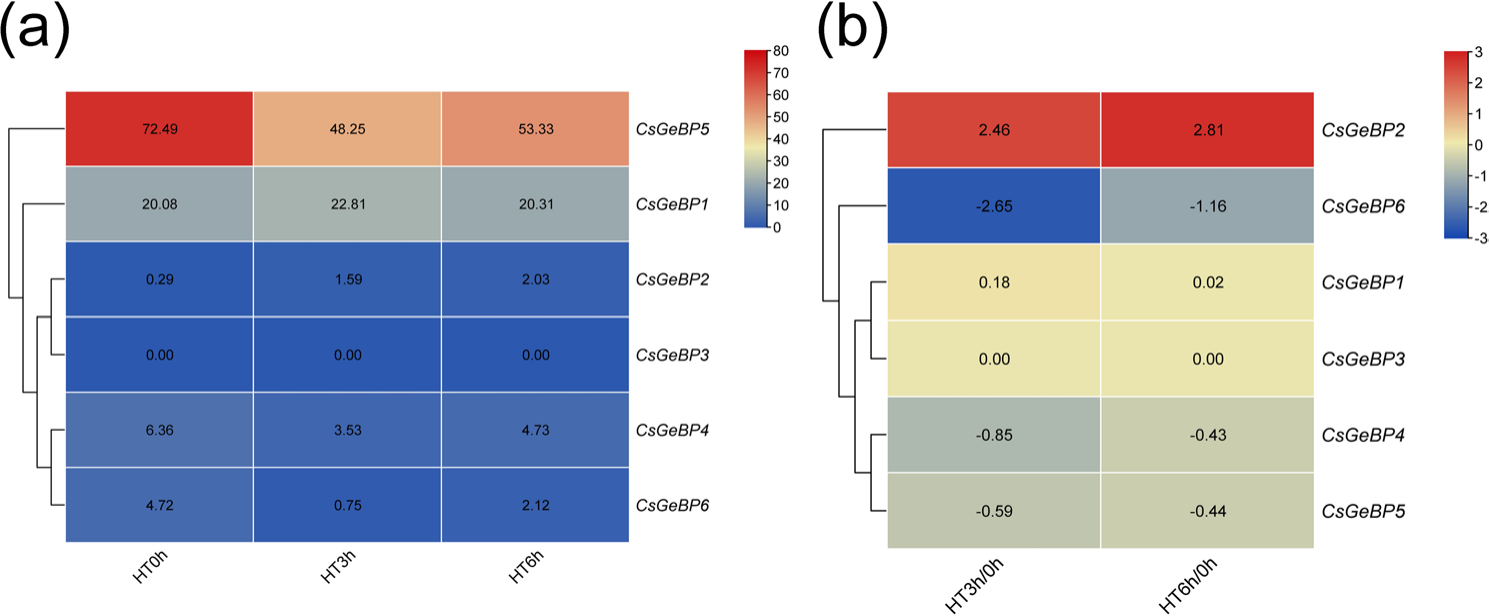
Expression heatmaps of *CsGeBP* genes Under heat stress. HT0h represents the control treatment, HT3h indicates high-temperature treatment for 3 h, and HT6h indicates high-temperature treatment for 6 h. **a** The data in the table represent the raw FPKM values. **b** The data in the table represent the log2 FC values

**Fig. 10:**
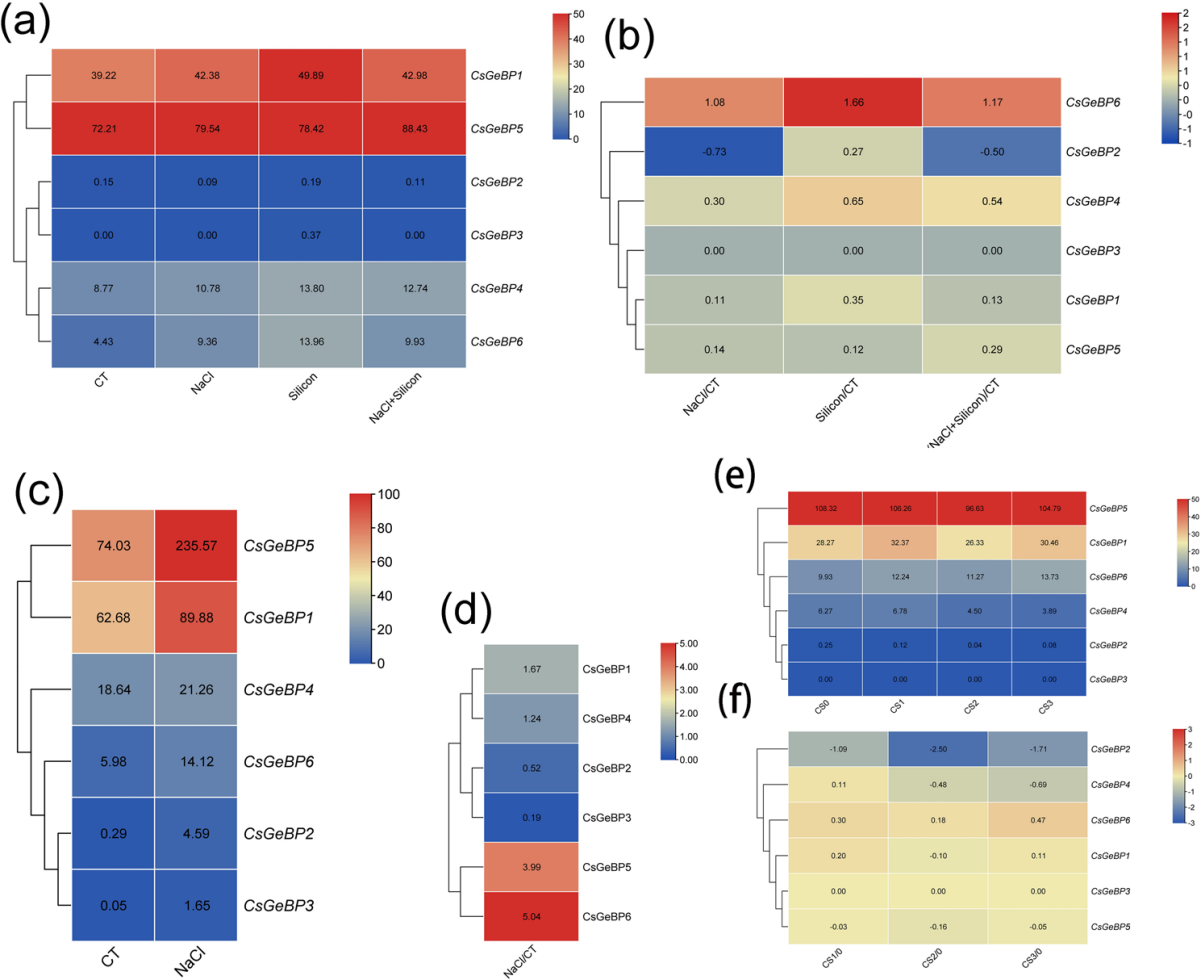
Expression heatmaps of *CsGeBP* genes Under stress. **a**, **b** Under NaCl and Si stress. **c**, **d** Under NaCl stress. **e**, **f** Under low-temperature stress. **a**, **c**, **e** The data in the table represent the raw FPKM values. **b**, **d**, **f** The data in the table represent the log2 FC values

Under heat stress, *CsGeBP2* was strongly upregulated after 3 h and 6 h of heat treatment. In contrast, although *CsGeBP6* exhibited a low basal expression level, it was markedly downregulated under heat stress, with the strongest repression observed at 3 h (Fig. 9). These results indicate distinct transcriptional responses of *CsGeBP* family members to heat stress, suggesting that *CsGeBP2* may be involved in the heat stress response in cucumber. However, under low-temperature stress, cucumber *CsGeBP* genes did not exhibit significant differential expression with increasing duration of cold treatment (Fig. 10e and f).

Under salt stress, *CsGeBP1* and *CsGeBP5* displayed relatively high expression levels in both transcriptome datasets (PRJNA437579 and PRJNA477930); however, their expression changes were not consistently significant. In contrast, although *CsGeBP6* showed a low basal expression level, it was significantly upregulated relative to the control, with a consistent induction pattern observed in both datasets (Fig. 10a, b, c and d). Moreover, *CsGeBP6* expression was markedly induced under silicon (Si) treatment (Fig. 10a and b).

After infection with Downy Mildew (Fig. S1 a, b) or Southern root-knot nematode (Fig. S1 c, d), *CsGeBP5* consistently showed high expression in both resistant and susceptible cucumber lines; however, no significant differences were detected between lines or between pre- and post-inoculation. In contrast, the other *CsGeBP* genes exhibited relatively low expression levels during infection.

**Fig. 11:**
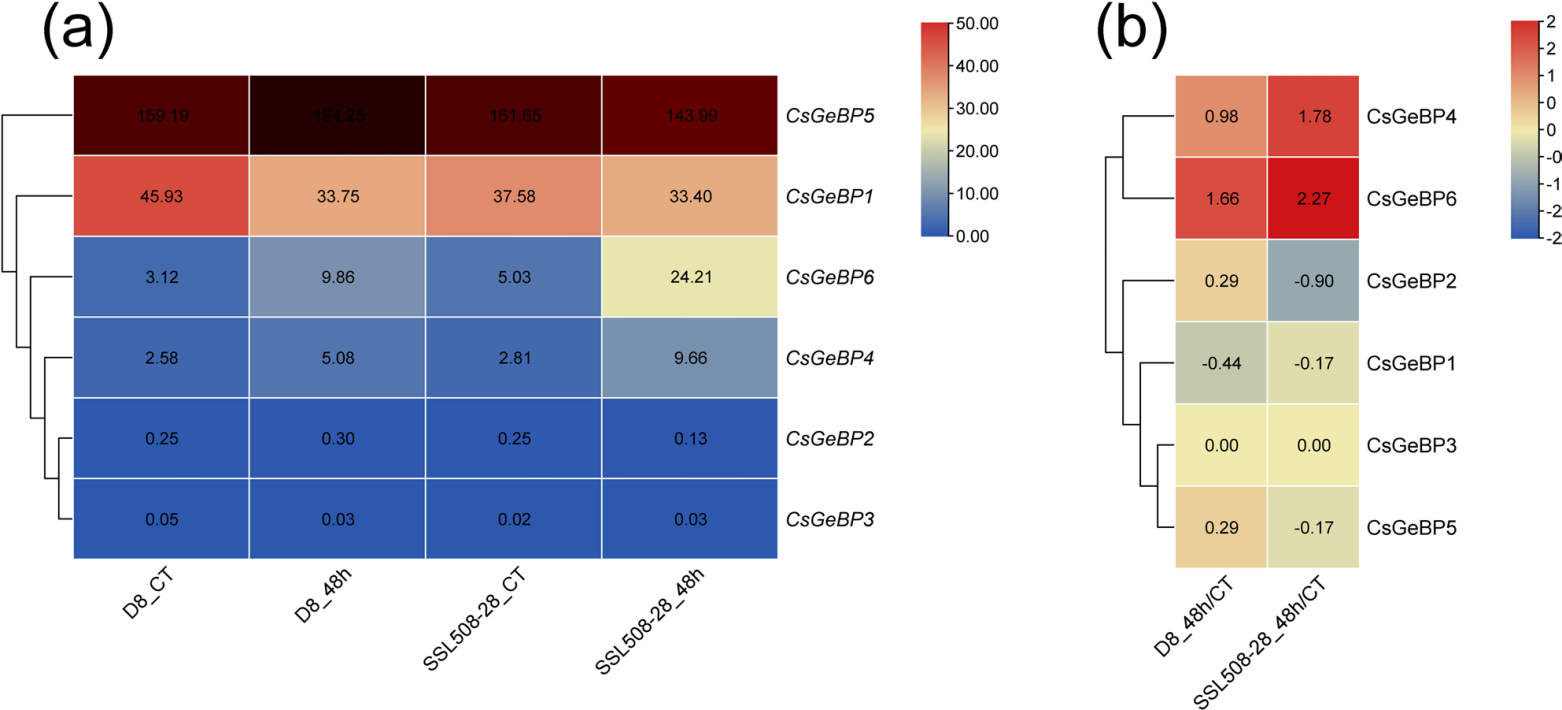
Expression heatmaps of *CsGeBP* genes under powdery mildew treatment. SSL508-28: resistant material; D8: susceptible material; CT: uninfected; 48 h, 48 h after inoculation. **a** The data in the table represents the raw FPKM values. **b** The data in the table represents the log2 FC values

At 48 h after powdery mildew inoculation, *CsGeBP4* and *CsGeBP6* were markedly induced in the resistant cucumber line SSL508-28, with expression levels significantly higher than those observed in the susceptible line D8, particularly for *CsGeBP6* (Fig. 11).

### The response of *CsGeBP6* under salt stress

**Fig. 12:**
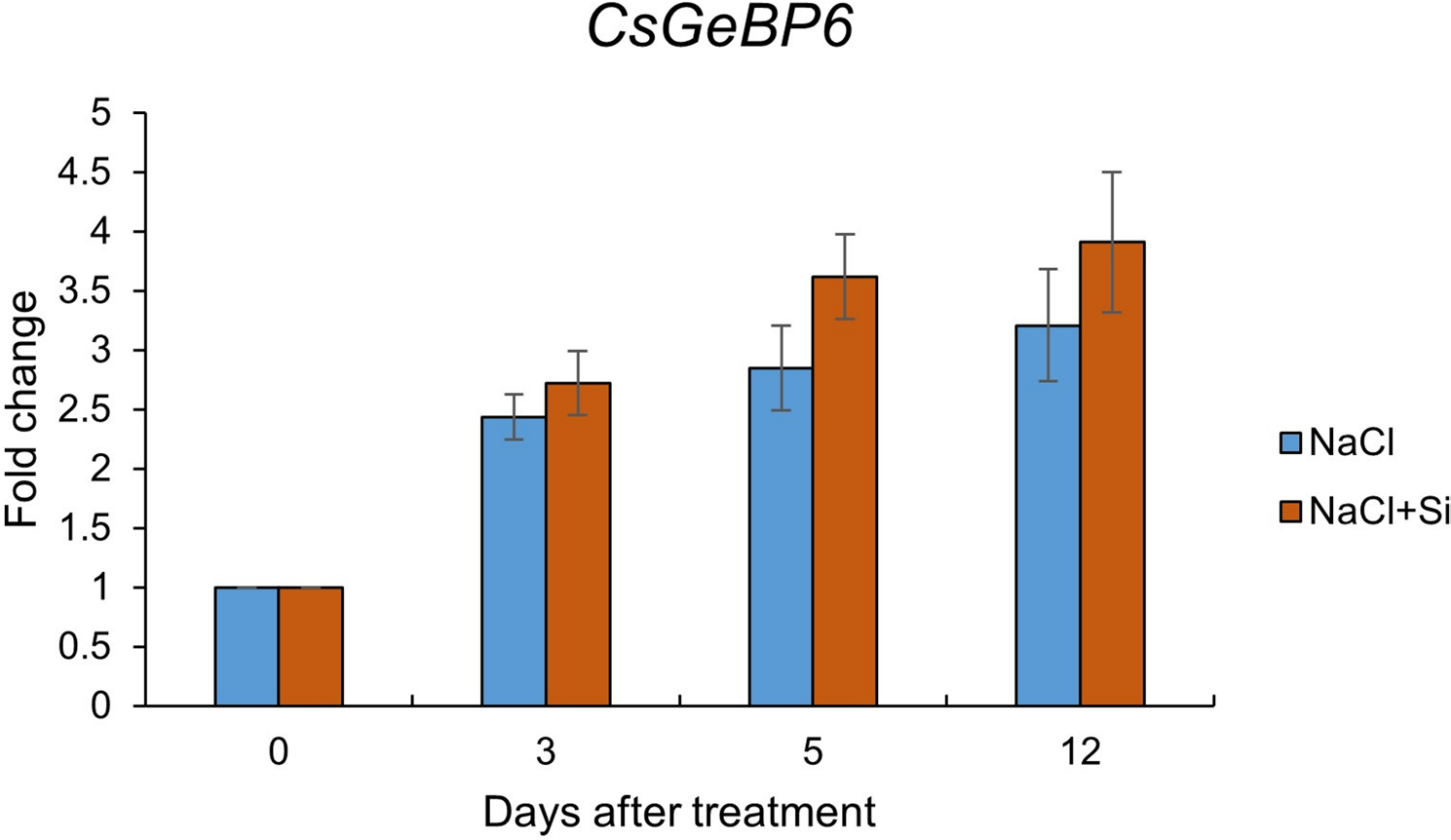
Expression of *CsGeBP6* salt stress treatment. NaCl: plants treated with 75 mM NaCl; NaCl + Si: plants treated with 75 mM NaCl supplemented with 0.5 mM organic silicon. The error bars in the graphs indicate SD. The value of relative expression showed the FC compared with that of the control

qRT-PCR analysis showed that *CsGeBP6* expression was markedly induced by salt stress (Fig. 12). Under 75 mM NaCl treatment, *CsGeBP6* transcript levels increased gradually over time, exhibiting a clear time-dependent upregulation. Moreover, *CsGeBP6* expression under the NaCl+silicon treatment was consistently higher than that under NaCl treatment alone at all examined time points, indicating that silicon application further enhanced the salt-induced expression of *CsGeBP6*.

## Discussion

In the present study, six *GeBP* family members were identified in the cucumber genome. This number is comparable to those reported in other dicotyledonous species, such as melon and watermelon, but is substantially lower than that observed in monocotyledonous plants, including wheat and rice [4]. Consistent with recent multi-species comparative genomic analyses, these results suggest that the *GeBP* gene family has maintained a relatively stable size in dicots, whereas a pronounced expansion has occurred in monocots [33]. Such lineage-specific differences in family size are likely attributable to whole-genome duplication events and divergent selective pressures following the divergence of monocot and dicot lineages [34].Phylogenetic analysis indicates that Group I likely represents the ancestral and most conserved subgroup of the *GeBP* family, as it includes members from all analyzed species and shows evidence of gene family expansion through multiple paralogous pairs (Fig. 4). Conversely, the exclusive presence of Group V and VI members in monocots supports the notion of pronounced lineage-specific evolution within the *GeBP* family after the monocot-dicot divergence [35]. Synteny analysis revealed that the expansion of cucumber *GeBP* genes primarily originated from segmental duplication rather than tandem duplication, which is highly consistent with the prevalent whole-genome duplication (WGD) events in the genomic evolutionary history of *Cucurbitaceae* [36]. The greater number of syntenic *GeBP* gene pairs identified between cucumber and melon also reflects their closer evolutionary relationship.

It has been reported that *GeBP* genes are involved in the development of fruit organs in plants. A genome-wide association study (GWAS) identified *GhGeBP4* as associated with fiber micronaire and strength, while *GhGeBP9* was linked to the node of the first fruiting branch and flowering time [37]. In this study, *CsGeBP5* shows sustained high expression in multiple vegetative and reproductive organs, with particularly high expression in ovaries and tendrils, suggesting their potential involvement in regulating both reproductive development and vegetative growth in cucumber. Recent studies on the regulatory network of fruit development have pointed out that *GeBP* genes play regulatory roles in fruit morphogenesis by integrating hormonal signals and cell expansion processes [1, 2, 38, 39]. Comparative analysis revealed that *CsGeBP5* and *CsGeBP1* are upregulated in long-fruit cucumber lines, while *CsGeBP3* and *CsGeBP6* exhibit marked expression differences, suggesting its potential involvement in the regulation of fruit length. Consistent with the findings of Cao et al. [40], *SlGeBP3*, *SlGeBP4*, *SlGeBP10*, and *SlGeBP11* show an expression pattern that peaks at the immature and mature green fruit stages before declining during ripening, indicating a likely function in fruit development.

Besides being involved in plant growth and development, *GeBP* genes also actively respond to both biotic and abiotic stresses. For example, the *Arabidopsis thaliana* transcription factor *GeBP-LIKE 4* (*GPL4*) inhibits root growth in response to toxic metal exposure by modulating reactive oxygen species levels, thereby allowing roots to preferentially colonize uncontaminated regions of the rhizosphere [41]. Our results showed that *CsGeBP6*, was markedly upregulated in response to salt stress, with a consistent induction observed in both datasets. Additionally, *CsGeBP6* expression was notably enhanced by silicon (Si) treatment. Previous studies have also suggested that silicon treatment acts as a stress elicitor. *CsGeBP6* may represent a candidate component of the silicon-mediated signaling pathway and is predicted to contribute to enhanced salt tolerance following its upregulation [28]. These results suggest that *CsGeBP6* may be involved in the cucumber’s salt stress response, and that Si treatment may further enhance its expression, potentially improving salt tolerance. Additionally, studies have also shown that the *GeBP* genes play a potential role in plant responses to pathogens [9, 32–43]. Our results indicate that, at 48 h post-inoculation with powdery mildew, *CsGeBP6* was significantly induced in the resistant line SSL508-28, with expression levels much higher than those observed in the susceptible line D8. This suggests that *CsGeBP6* could potentially participate in activating defense responses against powdery mildew. Similar studies have also been reported in wheat [44]. However, the specific mechanisms remain unclear. It is possible that *CsGeBP6* interacts with other defense-related transcription factors or is part of a larger gene regulatory network that enhances the plant’s ability to resist pathogen invasion, which need further functional characterization.

## Conclusions

This study provides the first systematic identification and multi-dimensional functional analysis of the cucumber *GeBP* gene family, revealing its potential roles in evolution, tissue development, and responses to abiotic and biotic stresses. Notably, *CsGeBP5* and *CsGeBP6* exhibit distinct expression patterns associated with developmental regulation and stress responsiveness, highlighting them as promising candidates for future functional validation and molecular breeding applications. Future studies integrating gene editing, transgenic approaches, and multi-omics analyses will facilitate deeper dissection of the regulatory mechanisms underlying *CsGeBP*-mediated stress adaptation and support the development of precision breeding strategies in cucumber.

## Supplementary Information

Supplementary Material 1. Additional file 1: Table S1. The entry ID for all genes discussed in this work. This work identified 77 GeBP gene family members in eghit crops. The gene names and entry ID of different species were listed.

Supplementary Material 2. Additional file 2: Figure S1. Expression heatmaps of CsGeBP genes Under stress. (a, b) infection with Downy Mildew. (c, d) infection with Southern root-knot nematode. (a, c) The data in the table represent the raw FPKM values. (b, d) The data in the table represent the log2 FC values.

## Data Availability

The authors confirm that the data supporting the findings of this study from NCBI database with accession numbers of PRJNA80169 (https://www.ncbi.nlm.nih.gov/bioproject/?term=PRJNA80169), PRJNA258122 (https://www.ncbi.nlm.nih.gov/bioproject/?term=PRJNA258122), PRJNA634519 (https://www.ncbi.nlm.nih.gov/bioproject/?term=PRJNA634519), PRJNA438923 (https://www.ncbi.nlm.nih.gov/bioproject/?term=PRJNA438923), PRJNA477930 (https://www.ncbi.nlm.nih.gov/bioproject/?term=PRJNA477930), PRJNA321023 (https://www.ncbi.nlm.nih.gov/bioproject/?term=PRJNA321023), PRJNA437579 (https://www.ncbi.nlm.nih.gov/bioproject/?term=PRJNA437579), PRJNA285071 (https://www.ncbi.nlm.nih.gov/bioproject/?term=PRJNA285071) and PRJNA419665 (https://www.ncbi.nlm.nih.gov/bioproject/?term=PRJNA419665) are available within the manuscript.

## Abbreviations

GeBPs: Glabrous Enhancer Binding Proteins
GL1: GLABROUS1
MFS: Major Facilitator Superfamily
PHT1: Phosphate Transporter Genes
HMM: Hidden Markov Model
CDD: Conserved Domain Database
WGD: Whole-Genome Duplication
GWAS: Genome-Wide Association Study
