# Supplementary figures and images for: Comprehensive analysis of the GeBP gene family in cucumber highlights functional divergence in fruit development and stress tolerance
Source: BMC Plant Biol. 2026 Apr 18;26:931. doi: 10.1186/s12870-026-08774-6 (PMC13224671; doi:10.1186/s12870-026-08774-6)

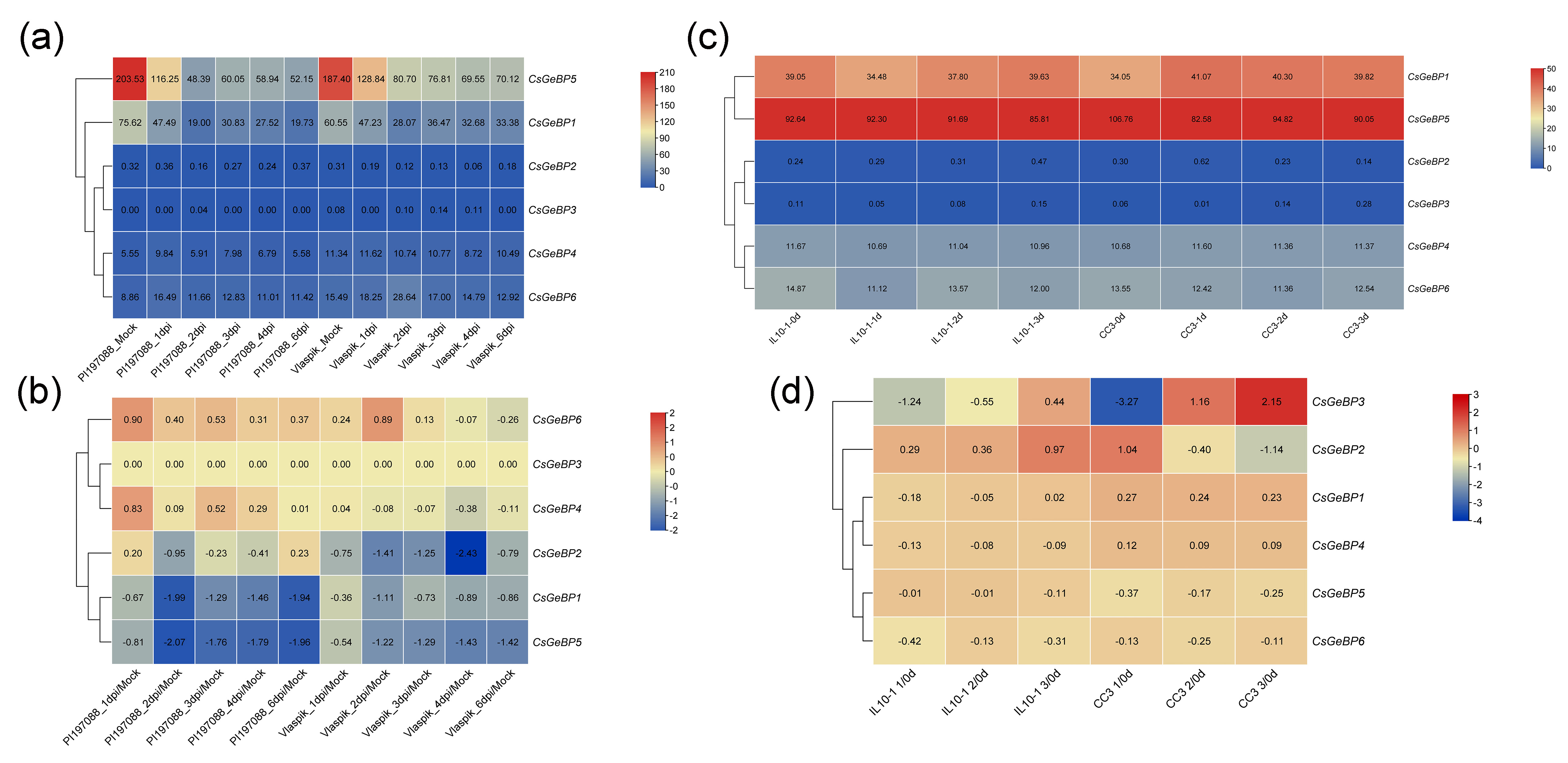

Supplement: Supplementary file 2 — Supplementary Material 2. Additional file 2: Figure S1. Expression heatmaps of CsGeBP genes Under stress. (a, b) infection with Downy Mildew. (c, d) infection with Southern root-knot nematode. (a, c) The data in the table represent the raw FPKM values. (b, d) The data in the table represent the log2 FC values. [file 12870_2026_8774_MOESM2_ESM.jpg]
